# Haploidentical Transplant in Radiosensitive Severe Combined Immunodeficiency Disease

**DOI:** 10.7759/cureus.45159

**Published:** 2023-09-13

**Authors:** Manpreet Saini, Alka R Khadwal, Sayan S Roy, Vignesh Pandiarajan, Pankaj Malhotra

**Affiliations:** 1 Clinical Hematology & Medical Oncology, Postgraduate Institute of Medical Education and Research, Chandigarh, IND; 2 Pediatric Allergy and Immunology, Advanced Pediatric Center, Postgraduate Institute of Medical Education and Research, Chandigarh, IND

**Keywords:** radiosensitive severe combined immunodeficiency disease, haploidentical transplant, severe combined immunodeficiency disease, non-homologous end joining gene, acute graft vs host disease, primary immunodeficiency disease, hematopoietic stem cell transplantation (hsct), scid

## Abstract

Severe combined immunodeficiency (SCID) is an inborn error of immunity invariably resulting in mortality in infancy until managed by hematopoietic stem cell transplant (HSCT). We present an unusual case of SCID with a rare mutation involving the non-homologous end-joining 1 (NHEJ1) gene, where a haploidentical HSCT was carried out with modified conditioning and graft versus host prophylaxis regimen using proteasome inhibitor bortezomib with a successful outcome.

## Introduction

Severe combined immunodeficiency disease (SCID) is a congenital immune disorder afflicting roughly one in 50,000 births [1]. Regrettably, the diagnosis is frequently missed in developing and underdeveloped countries due to limited awareness and diagnostic resources. Severe combined immunodeficiency disease is characterized by impaired T- and B-cell functionality right from birth. Among congenital immunodeficiencies, it represents the most severe form, often culminating in mortality by the age of two years unless immunologic reconstitution is accomplished via bone marrow transplantation [1].

It has various phenotypes, such as T-B+natural killer cells (NK cells)+, T-B-NK-, T-B+NK-, and T-B-NK+. During the process of generating diverse T- and B-cell receptor repertoires, double-strand DNA breaks are produced. One such process to correct these breaks is led via the non-homologous end-joining (NHEJ) system. Mutations in the genes involved in this process, such as the NHEJ1 gene, can cause a type of SCID that is characterized by immunodeficiency along with neurodevelopmental disease and sensitivity to ionizing radiation. An adjusted hematopoietic stem cell transplant is the safest method to ensure full T-cell immune reconstitution [2]. Infants with SCID are prey to severe infections such as diarrhea, sepsis, pneumonia, otitis media, cutaneous manifestations, and opportunistic infections [3]. Here, we report a case of a nine-month-old with SCID of the NHEJ1 variety who underwent a haploidentical transplant and was fully engrafted without any organ damage by using a modified conditioning regimen.

## Case presentation

A nine-month-old girl, born to parents who share a fourth-degree consanguineous marriage, presented with recurrent pneumonia and failure to thrive. This led to her being hospitalized twice previously. She was born at 36 weeks with a birth weight of 1.7 kg and was on exclusive breast feeds. After her third episode of pneumonia, she was evaluated, and her investigations are mentioned in Table 1 and Table 2.

**Table 1:** The patient's lab investigations

Test name	Results	Units	Reference interval
Hemoglobin	10	g/dl	11.10- 14.10
Total leucocyte count	1.80	thou/mm^3^	6.00-18.00
Platelet count	503	thou/mm^3^	200.00-550.00
Differential leukocyte count			
Neutrophils	22.30	%	14.00-55.00
Lymphocytes	40.90	%	37.00-79.00
Monocytes	24.50	%	2.00-12.00
Eosinophils	12.20	%	0.00-6.00
Basophils	0.10	%	0.00-1.00
Absolute leukocyte count			
Neutrophils	0.40	thou/mm^3^	1.00-6.00
Lymphocytes	0.74	thou/mm^3^	4.00-12.00
Monocytes	0.44	thou/mm^3^	0.20-1.20
Eosinophils	0.22	thou/mm^3^	0.10- 1.00
Basophils	0.00	thou/mm^3^	0.02-0.10

**Table 2:** The patient's serum immunoglobulin profile

Test name	Results	Units	Reference interval
Immunoglobulin G (IgG)	<140.00	mg/dl	220.00-900.00
Immunoglobulin M (IgM)	21.80	mg/dl	35.00-125.00
Immunoglobulin A (IgA)	<15.00	mg/dl	15.00-80.00

With the presence of lymphopenia and hypogammaglobulinemia, she was suspected to have an immunodeficiency disorder and was referred to our facility for genetic workup and management.

At the time of presentation to the pediatric immunology department, she showed signs of poor growth despite adequate feeding. Her height, weight, and occipitofrontal circumference were below the third percentile, and she had difficulty breathing due to persistent pneumonia, which was treated with antibiotics. A week later, she was detected to have cytomegalovirus viremia and screening negative for cytomegalovirus (CMV) retinitis. Thereafter, she received 14 days of intravenous (IV) ganciclovir along with IV immunoglobulin (IVIg), followed by oral valganciclovir till her viremia disappeared.

Her lymphocyte subset showed absolute CD3+ T lymphocytes as 173/cumm (2500-5600), CD19+ B lymphocytes as 58/cumm (430-3000), and CD56+ to dim+ NK cells as 821/cumm (70-830), which was suggestive of the T(-) B(-) NK(+) type of SCID. After next-generation sequencing was performed, she was diagnosed with SCID of the NHEJ gene {exon 2, c.169C>T, p.Arg57Ter} VAF-99.93% (homozygous, radiosensitive) variety and was started on a three-weekly immunoglobulin replacement regimen. Thereafter, she was referred to our bone marrow transplant center for a stem cell transplant. The father was chosen as a haplo-matched donor who was sex and major ABO-mismatched but a CMV-matched stem cell donor.

Peri-transplant course 

She received a conditioning regimen comprising fludarabine 150 mg/m2 over five days (day -7 to day -3), cyclophosphamide 20 mg/kg divided over two days (day -2 and day -1), and rabbit anti-thymocyte globulin 5 mg/kg over three days (day -7 to day -5) as per European Society for Blood and Marrow Transplantation (EBMT) protocol F [4]. In order to avoid possible complications of radiosensitivity, the conditioning regimen did not include irradiation, and low doses of alkylating agents were given. She developed two episodes of febrile neutropenia. The blood culture was positive for *Acinetobacter baumannii*, and she was treated as per antibiotic sensitivity. The fever settled on day -2, and granulocyte colony-stimulating factor (GCSF) mobilized 11.6 × 106/kg CD34+ peripheral blood stem cells were infused into the patient uneventfully. Bortezomib 1.3 mg/m2 (+6 and +48 hours), posttransplant cyclophosphamide (PTCy) 5 mg/kg on days +3 and +4, cyclosporine (CSA) 3 mg/kg/day, and mycophenolate mofetil 15 mg/kg three times a day from day +5 was given for graft-versus-host disease (GVHD) prophylaxis. The GCSF was started on day +5 and continued until neutrophilic engraftment happened.

Post-transplant course

The patient developed grade 1 cytokine release syndrome from day +2, which was associated with diarrhea that responded to empirical antibiotics (cefepime, metronidazole, and amikacin) by day +9. Neutrophil engraftment and platelet engraftment occurred on days +10 and +13, respectively. On day +16, she developed a central line-associated bacterial infection (*Staphylococcus haemolyticus*), which was treated with vancomycin, and the central line was salvaged. Post-transplant, the patient was continued on a three-weekly IVIg. Her lymphocytic immune reconstitution is mentioned in Table 3.

**Table 3:** Lymphocyte cells assay at baseline and post the HSCT * normal range WBC: white blood cells; NKT cells: natural killer T cells; HSCT: hematopoietic stem cell transplant

Day	Total WBC/mm^3 ^(6000-17500)*	Absolute lymphocyte/mm^3^ (3600-8900)*	CD3+T lymphocyte/mm^3 ^ (2500-5600)*	CD19+B lymphocyte/mm^3^ (430- 3000)*	CD56+NKT cells/mm^3^ (70-830)*
Baseline	2160	1140	173	58	821
Day +30	5600	2576	2498	6	66
Day +60	7810	3060	2802	146	73

Blood chimerism analysis showed 100% donor cells on day +30 as well as on day +60. She suffered from grade 3 skin GVHD on Day +47 but recovered after a course of steroids by day +60, and cyclosporin was switched to sirolimus prophylaxis as she was developing CSA toxicity in the form of hypertension. She had only grade 1 liver toxicity in the form of raised serum glutamic oxaloacetic transaminase (88 U/L, two times raised above the upper limit of normal 40 U/L) lasting for 48 hours only while bilirubin remained normal. Her creatinine and urea remained within normal limits until her discharge, as well as on subsequent follow-ups.

## Discussion

The incidence of SCID in India is not known [5]. Based on 10-year data from the Sample Registration System in India [6], Vignesh et al. have reported an approximate incidence of SCID at 0.12 per 100,000 live births out of 221 million live births [7]. While their study encompassed data from several centers responsible for SCID patient care in India, it’s important to note that the estimated incidence derived from this research might not accurately represent the true national incidence. This is due to the fact that not all the centers were included in their study.

The majority of primary immunodeficiency diseases (PIDs) follow an autosomal recessive pattern, making them more prevalent in regions where consanguineous marriages are frequent. Reports concerning PIDs from these regions have revealed a distinct prevalence of more severe disease manifestations in comparison to other areas. The increased severity is linked to elevated rates of both morbidity and mortality [8]. There are various types of SCID, and out of those, the non-homologous end-joining gene 1 (NHEJ1) defect is a rare form. Very few cases of this SCID variety have been described around the world. The ubiquitous absence of enzymes necessary for repairing DNA double-strand breaks leaves patients vulnerable to the consequences of immunodeficiency. This leads to increased susceptibility to infection, bone marrow failure, and the development of malignancies [9].

Classically, these patients are not infected immediately after birth. However, in the absence of an early diagnosis, they will eventually present with severe, atypical, or recurrent life-threatening infections. In addition to opportunistic infections, these patients also commonly suffer from non-specific bacterial infections [1]. This finding was also observed in our patient, who presented with recurrent episodes of pneumonia, which ultimately led to a timely referral to our center.

Hematopoietic cell transplantation (HCT) is curative in patients with SCID, but co-administered chemotherapy or radiotherapy is damaging in the NHEJ SCID subtype because of defective reparative mechanisms. Considering the heightened cellular radiosensitivity of our patient, we adopted a conditioning regimen in line with the EBMT protocol F, where alkylating agents are used in doses lower than conventional doses to avoid early organ toxicity and transplant-related mortality [4]. It is similar to the protocol used by McCloy et al. [10], where conditioning consisted of fludarabine (30 mg/m2/day for four days), cyclophosphamide (10 mg/kg/day for four days), and anti-thymocyte globulin (ATG) (15 mg/kg for four days). In our patient, the cyclophosphamide dosage was minimized, amounting to 20 mg administered over the course of two days.

In a study by Slack et al. [11], the outcomes of HSCT were examined in a cohort of 87 cases. Among these, 17 cases (19.5%) exhibited NHEJ mutations. The study employed reduced-intensity conditioning (RIC) in 59 cases, involving either no alkylators and/or a maximum of 150 mg/m2 fludarabine, along with 40 mg/kg of cyclophosphamide. Meanwhile, 22 cases received myeloablative conditioning (MAC). The results indicated that patients subjected to MAC had a significantly lower survival rate of 41%, as compared to the 79% survival rate observed in those who received reduced-intensity conditioning (p = 0.006). The authors concluded that RIC hematopoietic stem cells resolve and have a better outcome for DNA repair disorder-associated immunodeficiency.

This case is unique in another aspect, as the GVHD prophylaxis received by the patient was also modified. A low-dose PTCy and bortezomib combination was used for our patient. As per the literature, dendritic cells (DC) hold a crucial role in the initial stages of GVHD development and are considered a promising target for preventive measures against GVHD prevention. Bortezomib has demonstrated its ability to impede the maturation and function of DCs, and it also exhibits several beneficial immunomodulatory effects [12]. Ahmad et al. [13] reported the combination of cyclophosphamide at a fixed dose (50 mg/kg on days +3 and +4) and bortezomib on days 0 and +3 for GVHD prophylaxis in patients undergoing matched donor allogeneic transplant in hematological malignancies. The combination was well tolerated by all the patients, and it also allowed timely engraftment. The incidences of acute GVHD reported were 20% in grades II to IV and 6.7% in grades III and IV. The projected two-year disease-free survival and overall survival were 55.7% and 68%, respectively. The study concluded that the use of bortezomib is a practical approach for allotransplants, aligning with the approach taken in our patient’s case. Koreth et al. [14] conducted a study focused on a high-risk group that had undergone mismatched unrelated donor transplants. They studied the efficacy of combining bortezomib with methotrexate and tacrolimus as part of their treatment regimen. They found a reduction in GVHD along with an enhancement of immune reconstitution. We applied the concept of using bortezomib in combination with low-dose PTCy as a GVHD prophylaxis regimen in our index haplo-matched reduced-intensity allogeneic HSCT.

## Conclusions

To the best of our knowledge, this is the first detailed description of modified conditioning as well as GVHD prophylaxis regimen for haploidentical HSCT and of the clinical course after transplant in an NHEJ1 type of SCID. Considering the genetic defect observed in our patient with a predilection for heightened cellular radiosensitivity, a conditioning regimen involving total body irradiation and high-dose cyclophosphamide would likely not have been well-tolerated. It is notable that our patient achieved successful engraftment without any organ damage and is maintaining full donor chimerism until the time of submission of this manuscript.
